# Application of flow cytometry with a fluorescent dye to measurement of intracellular nitric oxide in plant cells

**DOI:** 10.1007/s00425-018-2901-2

**Published:** 2018-04-27

**Authors:** Jan Kępczyński, Danuta Cembrowska-Lech

**Affiliations:** Department of Plant Physiology and Genetic Engineering, Faculty of Biology, University of Szczecin, Wąska 13, 71-415 Szczecin, Poland

**Keywords:** DAF-FM DA, Flow cytometry, Fluorescence, Nitric oxide, Plant material, Plant stress

## Abstract

A simple and rapid method involving flow cytometry and NO-specific probe (DAF-FM DA) proved useful for detection and determination of intracellular NO production in *Medicago truncatula* suspension cells and leaves as well as in cells of *Avena fatua*, *Amaranthus retroflexus* embryos and leaves.

The measurement of nitric oxide (NO) in plant material is important for examining the regulatory roles of endogenous NO in various physiological processes. The possibility of detecting and determining intracellular NO production by flow cytometry (FCM) with 4-amino-5-methylamino-2′,7′-difluorofluorescein (DAF-FM DA), an NO-specific probe in *Medicago truncatula* cells in suspension and leaves as well as in cells of embryos and leaves of *Avena fatua* L. or *Amaranthus retroflexus* L. was explored. To detect and measure NO production by cell suspension or embryos and leaves, the recommended DAF-FM DA concentration is 5 or 10 µM, respectively, applied for 30 min. Exogenous NO increased the intensity of the fluorescent signal in embryos and leaves of both plants, while carboxy-PTIO (cPTIO), an NO scavenger, decreased it. Thus, these results demonstrate that NO can be detected and an increase and a decrease of its intracellular level can be estimated. Wounding was observed to increase the fluorescence signal, indicating an increase in the intracellular NO level. In addition, the levels of exogenous and endogenous ascorbic acid were demonstrated to have no effect on the NO-related fluorescence signal, indicating the signal’s specificity only in relation with NO. The applicability of the proposed method for detection and determination of NO was confirmed (1) by in situ NO imaging in cell suspensions and (2) by determining the NO concentration in embryos and leaves using the Griess reagent. In view of the data obtained, FCM is recommended as a rapid and simple method with which to detect and determine intracellular NO production in plant cells.

## Introduction

Nitric oxide (NO) is a small hydrophobic molecule with chemical properties that make it uniquely suitable as both an intra- and extracellular messenger (Neill et al. 2008). This gaseous molecule plays a role as a molecular mediator of a variety of physiological processes in plants, including breaking of seed dormancy, seed germination, seedling growth, flowering, senescence, and responses to biotic and abiotic stresses (Mur et al. 2013). To understand the physiological function of NO in plants, it is important to determine endogenous concentration of NO in plant tissue. However, the unstable nature of the NO molecule and its low level complicate the development of an appropriate method of NO detection and determination of its concentration. Despite these difficulties, several methods for the measurement of NO in plants have been developed, such as a spectrophotometric assay which determines the product of Griess reaction with nitrite azo dye (Tracey 1992) or the product of haemoglobin oxidation (Murphy and Noack 1994), laser-based infrared spectrometry (LAPD) (Mur et al. 2005), electron spin resonance (ESR) (Kleschyov et al. 2007) or voltametric assay with NO electrode (Davies and Zhang 2008). In addition, two techniques based on the use of fluorescent NO indicators, diaminofluoresceins (DAF_s_): DAF-2 DA, DAF-FM DA or DAF-FM, have been developed. These dyes can be used to detect NO by fluorescence microscopy or to determine its concentration by fluorimetry (Vitecek et al. 2008; Mur et al. 2011).

Extracellularly applied DAF-2 DA or DAF-FM DA spontaneously crosses the plasma membrane and is deacetylated by esterases to generate intracellular non-fluorescent DAF-2 or DAF-FM, respectively, which are then oxidised by NO-to-triazole products responsible for fluorescence (Kojima et al. 1998, 1999). DAF-2DA was found to be less sensitive to NO than DAF-FM; the NO detection limit was ca 5 and 3 nM, respectively (Murad 1999). The fluorescence dyes DAF-2 DA (Navarro-Antolin and Lamas 2001; Strijdom et al. 2004; Tiscornia et al. 2009) and DAF-FM (Paul et al. 2011) were also used to detect and measure intracellular NO in human and mammalian cells by flow cytometry (FCM). It was emphasized that unlike the fluorometric methods, FCM is a very sensitive method for detecting cells producing NO within the cell population (Tiscornia et al. 2009). Due to deficiencies inherent in various methods of NO determination in plant material and a suggestion to use more than one measurement technique in parallel, it is reasonable to develop or adapt another method (Vitecek et al. 2008; Mur et al. 2011). Until now, FCM has not been applied for the detection or measurement of NO in plant cells.

The aim of the present work was to adapt flow cytometry, using DAF-FM DA, to determine plant cell intracellular NO production. Application of FCM to the detection of nitric oxide in cells of *Medicago truncatula* Gaertn suspension and leaves as well as its measurement in cells of embryos and intact and wounded leaves of *Avena fatua* L. and *Amaranthus retroflexus* L. is presented. To check whether the fluorescence signal is associated with NO, fluorescence was measured after embryos and leaves were treated with exogenous NO and cPTIO, an NO scavenger. Furthermore, since diaminofluorescein DAF-2 was demonstrated to be reactive to ascorbic acid (AsA) in animal cells (Zhang et al. 2002), effects of exogenous AsA and lycorine (LYC), an inhibitor of its biosynthesis, on the fluorescence signal were followed. The usefulness of FCM with DAF-FM DA in NO detection and determination of its production was explored using a bioimaging system and the Griess reaction.

## Materials and methods

### Plant materials

#### Cell suspensions

Calluses were produced from leaf explants of *Medicago truncatula* cv. Jemalong, cultivated in a growth room at 24 ± 1 °C under a 16 h photoperiod of 70 µmol m^−2^ s^−1^ GreenLED (Philips), according to Orłowska et al. (2017). For callus induction, initial explants were placed on Petri dishes (ø 55 mm) with the SH medium (Schenk and Hildebrandt 1972) containing 0.5 µM 2,4-d (Duchefa Biochemie, Haarlem, The Netherlands) and 1.0 µM zeatin (Duchefa Biochemie) with 30 g l^−1^ sucrose solidified with 2.5 g l^−1^ gerlit and adjusted to pH 5.7. The cultures were kept at 28 ± 1 °C in the dark for 21 days. Suspension cultures were initiated from 0.5 g proliferated calluses by sub-culturing in 100 ml Erlenmeyer flasks in 25 ml liquid B_5_ medium (Gamborg et al. 1968) supplemented with 2.5 µM 2,4-d and 4.5 µM kinetin (Duchefa Biochemie) at 18 °C for 14 days.

#### Seeds, embryos and leaves

The experiments were carried out on wild oat (*Avena fatua* L.) and redroot pigweed (*Amaranthus retroflexus* L.) embryos isolated from non-dormant caryopses or partially non-dormant seeds, respectively or from leaves of 10-day-old seedlings of the species mentioned and 2-month-old *M. truncatula* plants. Caryopses of *A. fatua* and seeds of *A. retroflexus* were collected in 2013 and 2007, respectively, during the time of their natural dispersal, in the vicinity of Szczecin (Poland). After collection, the caryopses and seeds were dried at room temperature, for 7 days, to a constant moisture content (ca. 12%). To obtain non-dormant caryopses and partially non-dormant seeds, they were stored dry under ambient relative humidity for up to 4 months (*A. fatua*) or 3 weeks (*A. retroflexus*) in the dark at 25 °C.

### Seed, embryo, and leaf treatments

#### Seed treatment with lycorine

*Avena fatua* caryopses (25 in five replicates) or *A. retroflexus* seeds (50 in five replicates) were incubated in Petri dishes (ø 6 cm) on one layer of filter paper moistened with 1.5 ml of water or lycorine (LYC) (2 × 10^−4^ M) (Sigma-Aldrich) for 24 h in the dark at 20 °C or at 25 °C in the light (light intensity 120 µmol m^−2^ s^−1^; 16/8 h photoperiod), respectively. After 24 h, the embryos were isolated from imbibed seeds and used for the FCM, Griess or AsA assays.

#### Seed treatment with high temperature

*Avena fatua* caryopses (25 in five replicates) or *A. retroflexus* seeds (50 in five replicates) were dry-stored at 105 °C (air temperature) for 24 h. Subsequently, the caryopses and seeds were incubated in Petri dishes (ø 6 cm) on one layer of filter paper moistened with 1.5 ml of water for 24 h in the dark at 20 °C or at 25 °C in the light (light intensity 120 µmol m^−2^ s^−1^; 16/8 h photoperiod), respectively. After 24 h of incubation, the embryos isolated from untreated and treated caryopses and seeds were used for the FCM assay. To find out whether high-temperature-treated caryopses and seeds were dead, they were incubated in Petri dishes on filter paper moistened with 1.5 ml of water for 7 days. In contrast to the untreated caryopses and seeds, the treated ones were not able to germinate.

#### Embryo treatment with NO, cPTIO, and AsA

Five uncovered Petri dishes (ø 6 cm), each with five embryos of *A. fatua* or *A. retroflexus* on one layer of filter paper moistened with 1 ml of distilled water, NO scavenger carboxy-2-(4-carboxyphenyl)-4,4,5,5-tetramethylimidazoline-1-oxyl-3-oxide (cPTIO) (10^−3^ M) (Enzo Life Sciences) or ascorbic acid (AsA, 10^−7^, 10^−6^, 10^−3^ M) (Sigma-Aldrich) and one open Petri dish (ø 6 cm) containing water or 10^−2^ M KNO_2_ (NO donor) (5 ml 2 × 10^−2^ M KNO_2_ acidified with 5 ml 2 × 10^−1^ M HCl), prepared as described by Yamasaki (2000), were enclosed in a 19-cm (0.5 l) Petri dish, which was sealed with Parafilm. After incubation for 1 h at 20 °C in the dark (*A. fatua*) or 25 °C in the light (light intensity 120 µmol m^−2^ s^−1^) (*A. retroflexus*), the untreated and treated embryos were rinsed three times in 10 mM Hepes–KOH pH 7.4 prior to the FCM or Griess assay, and rinsed three times in sterile water before the AsA assay.

#### Leaf treatment with NO, cPTIO, and AsA

Five leaves (in five replicates) isolated from 10-day-old seedlings developed from non-dormant *A. fatua* caryopses and *A. retroflexus* seeds after incubation in water were placed in Petri dishes (ø 6 cm) on filter paper moistened with 1 ml of water or cPTIO (10^−3^ M) or ascorbic acid (10^−7^, 10^−6^, and 10^−3^ M). These dishes and one open Petri dish with water or 5 ml 2 × 10^−2^ M KNO_2_ (NO donor) with 5 ml 2 × 10^−1^ M HCl, were enclosed in a 19-cm (0.5 l) Petri dish and sealed with Parafilm. After incubation for 1 h at 20 °C (*A. fatua*) or 25 °C (*A. retroflexus*) in the light (light intensity 120 µmol m^−2^ s^−1^), the leaves were rinsed three times in 10 mM Hepes–KOH pH 7.4 before the FCM or Griess assay, and rinsed three times in sterile water before the AsA assay.

### Mechanical wounding

The experiment with mechanical wounding involved leaves of *A. fatua*, *A. retroflexus* and *M. truncatula*. A 5- or 2-mm-long peak fragment (three in five replicates) was cutoff from a leaf of *A. fatua* or *A. retroflexus*, respectively. The mechanically wounded leaves were placed in Petri dishes (ø 6 cm) on one layer of filter paper moistened with 1 ml of sterile water and incubated for different time at 25 °C in the light of 120 µmol m^−2^ s^−1^. In the next experiment with *M. truncatula*, three square-shaped explants (each in five replicates) 1 cm in side length were cutoff from each trifoliate leaf and incubated in Petri dishes (ø 6 cm) on one layer of filter paper moistened with 1 ml of sterile water for different period of time at 25 °C in the light of 70 µmol m^−2^ s^−1^ GreenLED. After 15, 30, 45, or 60 min, the leaves of all the species were rinsed three times in 10 mM Hepes–KOH pH 7.4 prior to the FCM or Griess assay, and were rinsed three times in sterile water before the AsA assay.

### DAF-FM DA staining

To measure the fluorescence signal related to DAF-FM and NO product present in the plant cells, a membrane-permeable form of dye, DAF-FM DA, was used. Cell suspensions (200 µl in five replicates) of *M. truncatula* were incubated in 5–30 µM DAF-FM DA (Sigma-Aldrich) in 10 mM Hepes–KOH pH 7.4 for 30 or 60 min at 25 °C in the dark. After incubation, the cells were washed three times in 10 mM Hepes–KOH pH 7.4. The treated and untreated embryos (5 in five replicates) or leaves (in five replicates) were washed and then incubated in 5–30 µM DAF-FM DA in 10 mM Hepes–KOH pH 7.4 for 30 or 60 min at 25 °C in the dark. Subsequently, the embryos or leaves were washed three times in 10 mM Hepes–KOH pH 7.4.

### Cell isolation and flow cytometry analysis

The stained *M. truncatula* cells from the suspension as well as embryos of *A. fatua* and *A. retroflexus* or leaves of these species were transferred to 2 ml of the isolation buffer, pH 7.4, containing 45 mM MgCl_2_, 30 mM sodium citrate, 20 mM MOPS and 0.1% (v/v) Triton X-100 (Galbraith et al. 1983) and, using a razor blade, chopped for 2 min. Subsequently, the suspension was passed through a 50 µm nylon mesh. The labelled cells were analyzed using a Partec PAII flow cytometer (Partec) with an air-cooled 20 mV argon-ion laser. In the flow chamber, each cell crossed a region of fluorescence excitation. There, the green-fluorescence (FL-III) emissions of DAF-FM T were induced consecutively with blue light at 488 nm and recorded by photomultiplier tubes (PMT5 RED) at 515 nm. The flow cytometer was equipped with dichroic mirror with the edge at 420 nm (TK420), a full mirror (FM) and a long pass filter with the edge at 515 nm (OG515). The final gated cell populations contained 20,000 cells and signals were recorded on a histogram by logarithmic amplifiers. The histograms depict the fluorescence intensity (log; Geo Mean) on the *x*-axis (FL-III) and cell counts in gated cell populations on the *y*-axis. The relative NO level was expressed as the mean fluorescence intensity (percentage of the control).

### Intracellular DAF-FM-triazole imaging

The intracellular NO location was analyzed with the aid of a real-time living cell imaging system. The *M. truncatula* cell suspension (200 µl in five replicates) was incubated in 5–30 µM DAF-FM DA (Sigma-Aldrich) in 10 mM Hepes–KOH pH 7.4 for 30 or 60 min at 25 °C in the dark. After incubation and washing three times in 10 mM Hepes–KOH pH 7.4, 50 µl of the suspension was transferred onto a standard 96-well plate with 200 µl of 10 mM Hepes–KOH pH 7.4. Cell imaging was performed with the aid of a BD Pathway 855 Bioimager system using *λ*_Ex/Em_ = 495/515 nm.

### The Griess assay

The total NO was determined in embryos of *A. fatua* and *A. retroflexus* as well as in leaves of these species and *M. truncatula* using modified method of Griess (Liu et al. 2007). Samples (50 mg fresh weight in five replicates) were homogenized in 40 mM Hepes pH 7.4. The homogenate was centrifuged for 20 min at 14,000*g* and 4 °C. The reaction mixture containing 80 µl of the supernatant and 100 µl of the Griess reagent was incubated at 25 °C for 15 min. The spectrophotometric measurement of the azo product absorbance was carried out at 540 nm for the supernatant preincubated for 30 min in the absence or presence of cPTIO (5 × 10^−4^ M), a specific scavenger of NO. The NO content was compared with the NaNO_2_ standard curve as a difference between samples preincubated in the absence or presence of cPTIO, and expressed as nmol g^−1^ FW.

### Ascorbic acid content measurement

The AsA content was determined in embryos of *A. fatua* and *A. retroflexus* and in leaves of these species and *M. truncatula*. The samples (50 mg fresh weight in five replicates) were homogenized for 10 min in 0.4 ml of 6% (w/v) TCA (Kampfenkel et al. 1995). The extracts were centrifuged at 15,000*g* for 20 min. The supernatant obtained (50 µl) or 6% (w/v) TCA was added to the reaction mixture containing 0.15 ml 0.2 M K-buffer pH 7.4, 0.05 ml ddH_2_O, 0.25 ml 10% (w/v) TCA, 0.2 ml 44% (v/v) H_3_PO_4_, 0.2 ml 4% (w/v) 2,2′-dipyridyl (Sigma-Aldrich) and 0.1 ml 3% (w/v) FeCl_3_. The reaction mixture was incubated for 40 min at 42 °C. The absorbance of the end product was measured at 525 nm using water as a reference. A standard curve was prepared with the AsA standard. The results are expressed as µmol AsA g^−1^ FW.

### Data analysis

The means (± standard deviations, SD) of five replicates are shown. Differences between the means were analyzed for significance using one-way analysis of variance, ANOVA (Statistica for Windows v. 12.0, Stat-Soft Inc., Tulsa, OK, USA). Duncan’s multiple range test was used to test for significance of differences (*P* ≤ 0.05) between relative NO levels. Two or five independent experiments produced similar results.

## Results

### DAF-FM-triazole fluorescence measured by flow cytometry

**Fig. 1:**
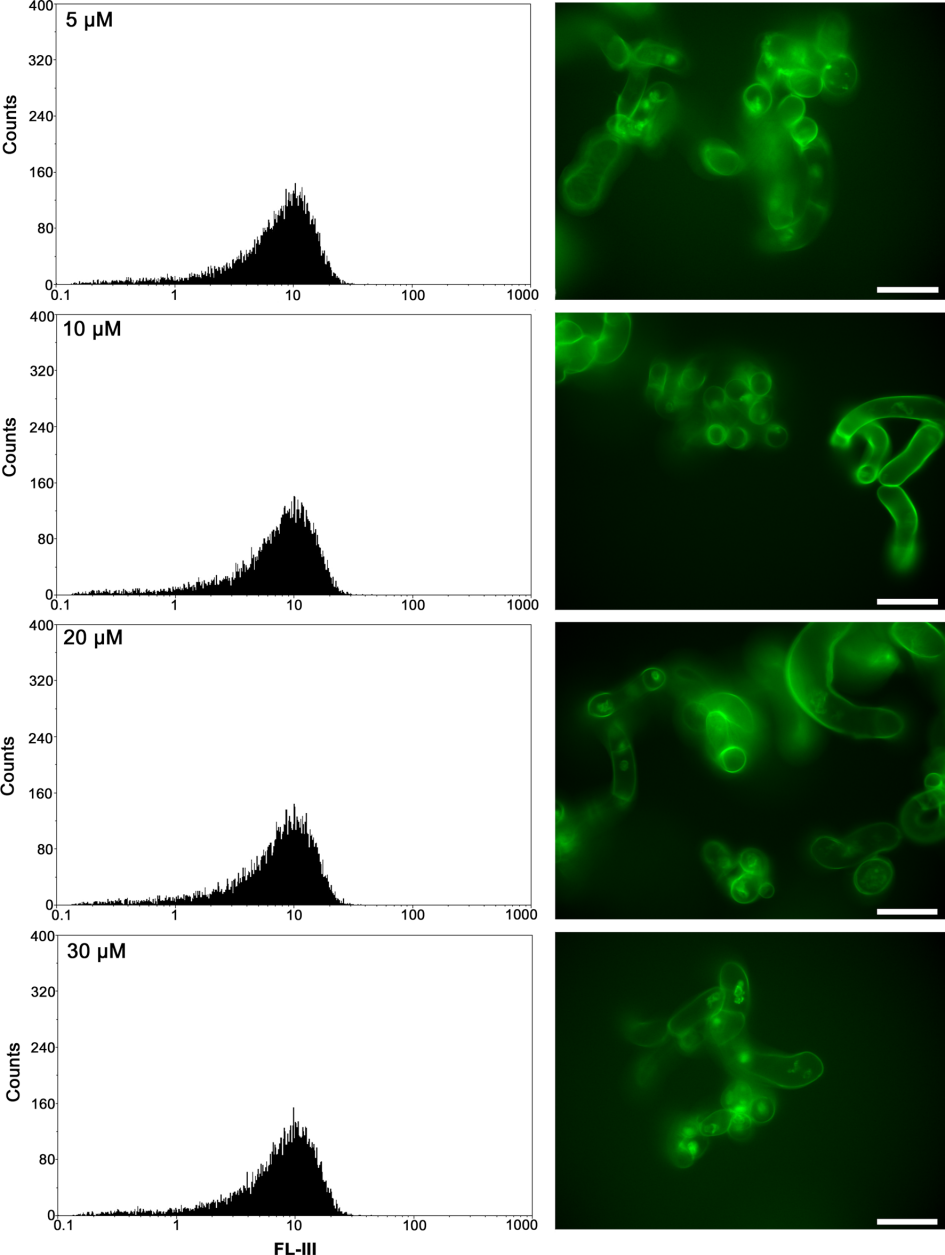
Flow data (left) and fluorescence images (right) of *Medicago truncatula* cell suspensions labelled with DAF-FM DA at different concentrations for 30 min. No autofluorescence was present in cells incubated without DAF-FM DA. Cells shown are representative images from replicate experiments (*n *= 5). Scale bar is equal to 50 µm

Intracellular NO was detected in the plant material by flow cytometry (FCM) using the NO-specific probe, 4-amino-5-methylamino-2′,7′-difluorofluorescein (DAF-FM DA). Following incubation of *Medicago truncatula* cell suspensions for 30 min with the dye mentioned at concentrations ranging from 5 to 30 µM, a fluorescence signal was detected, the signal corresponding to the presence of NO (Fig. 1). The fluorescence intensity remained stable regardless of the dye concentration used. Likewise, no significant differences were observed when 60 min incubation time was used (data not shown). Thus, the application of 5 µM DAF-FM DA for 30 min was sufficient to detect the NO level in cell suspensions.

**Fig. 2:**
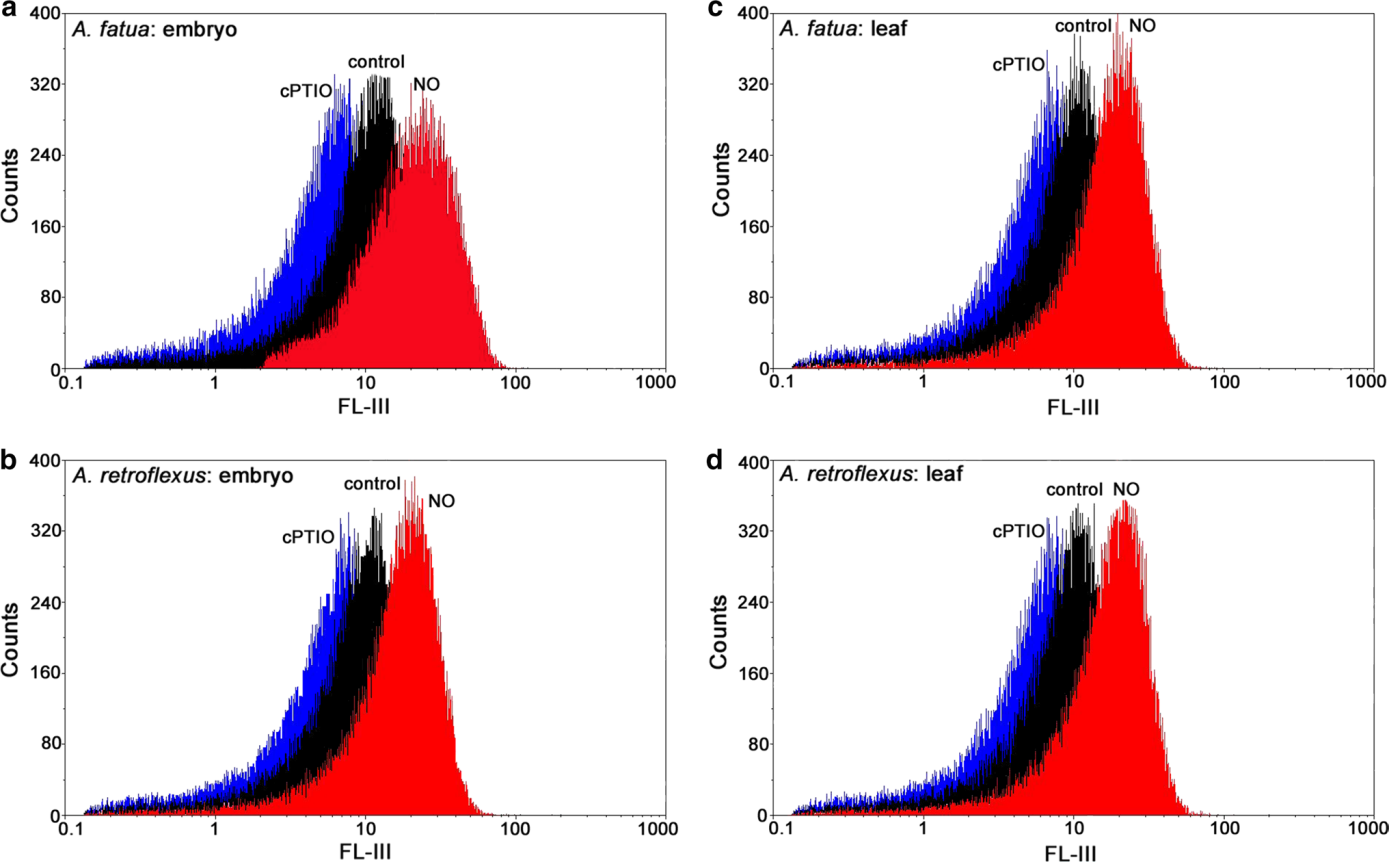
Flow data of *Avena fatua* (**a**, **c**) or *Amaranthus retroflexus* (**b**, **d**) embryos and leaves treated with DAF-FM DA at 10 μM for 30 min. Both the embryos and leaves were pretreated with NO or cPTIO for 1 h before incubation with DAF-FM DA. No autofluorescence was present when embryos or leaves were incubated without DAF-FM DA. Representative histograms show the fluorescence intensity (log; Geo Mean) on the *x*-axis (FL-III) and cell counts in gated cell populations on the *y*-axis (*n *= 5)

**Fig. 3:**
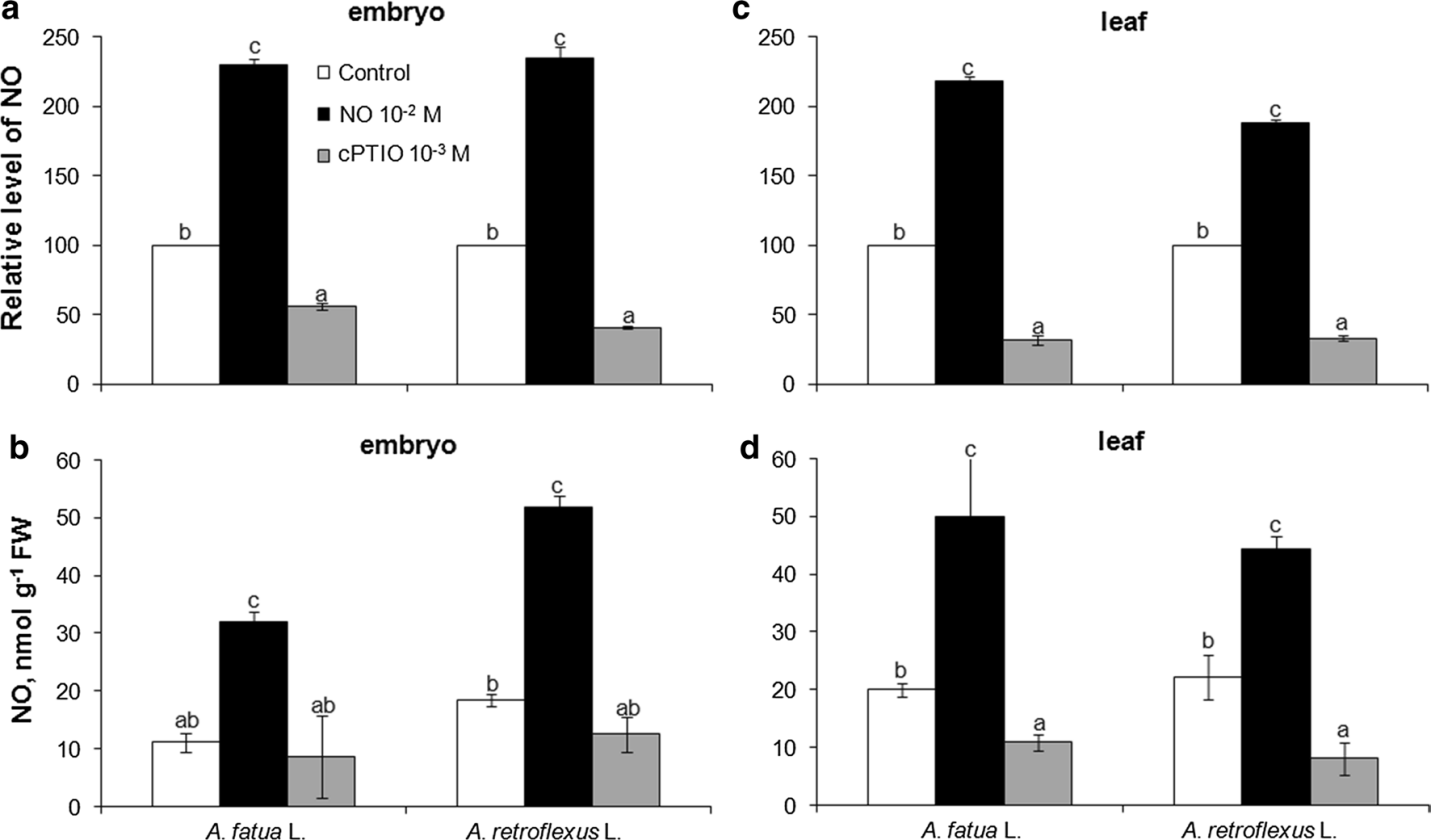
Effects of exogenous NO and cPTIO on the NO level as measured by FCM (**a**, **c**) or Griess reaction (**b**, **d**) in *A. fatua* and *A. retroflexus* embryos or leaves. Both embryos and leaves were pretreated with NO or cPTIO for 1 h before incubation with DAF-FM DA (FCM) or NO determination (Griess). Intracellular level of NO was measured by FCM after treatment with DAF-FM DA at 10 μM for 30 min. Fluorescence data are expressed as mean fluorescence intensity (percentage of control). No autofluorescence was present when embryos or leaves were incubated without DAF-FM DA. Vertical bars indicate ± SD. One-way ANOVA with Duncan’s post hoc test was used to identify significant differences. Means with different letters (a–c) are significantly different (*P* < 0.05)

The fluorescence level was also determined in cells of embryos and leaves of *Avena fatua* or *Amaranthus retroflexus*. As expected, the fluorescence signal was undetectable when embryos incubated in water were not stained with DAF-FM DA (not shown). Likewise, no fluorescence signal was detectable when dead embryos were treated with DAF-FM DA (not shown). When the dye was applied at 5 µM, no signal was detected, either. On the other hand, the fluorescence signal was measurable after incubation at 10–30 µM dye concentrations when embryos isolated from *A. fatua* caryopses and *A. retroflexus* seeds, previously imbibed in water, were used (Fig. 2a, b). The fluorescence observed after 10 µM DAF-FM DA application was similar to that produced when higher dye concentrations were used. The intensity of the signal obtained after 30 min incubation in the presence of the dye at 10–30 µM did not change when incubation was extended for up to 60 min (not shown). To find out if the fluorescence signal was triggered by the triazole fluorescein produced by the reaction between endogenous NO and DAF-FM, the embryos were incubated—prior to staining with DAF-FM DA—in the presence of NO for 1 h. Exogenous NO was found to increase the mean signal from embryos of *A. fatua* and *A. retroflexus* by a factor of 2.3, compared to the fluorescence signal from untreated embryos (Figs. 2a, b, 3a). In contrast, a marked decrease in fluorescence was observed when embryos were treated for 1 h with the NO scavenger cPTIO. Because of the cPTIO treatment, the fluorescence signal was by 1.8 and 2.4 times lower in embryos of *A. fatua* and *A. retroflexus*, respectively.

**Fig. 4:**
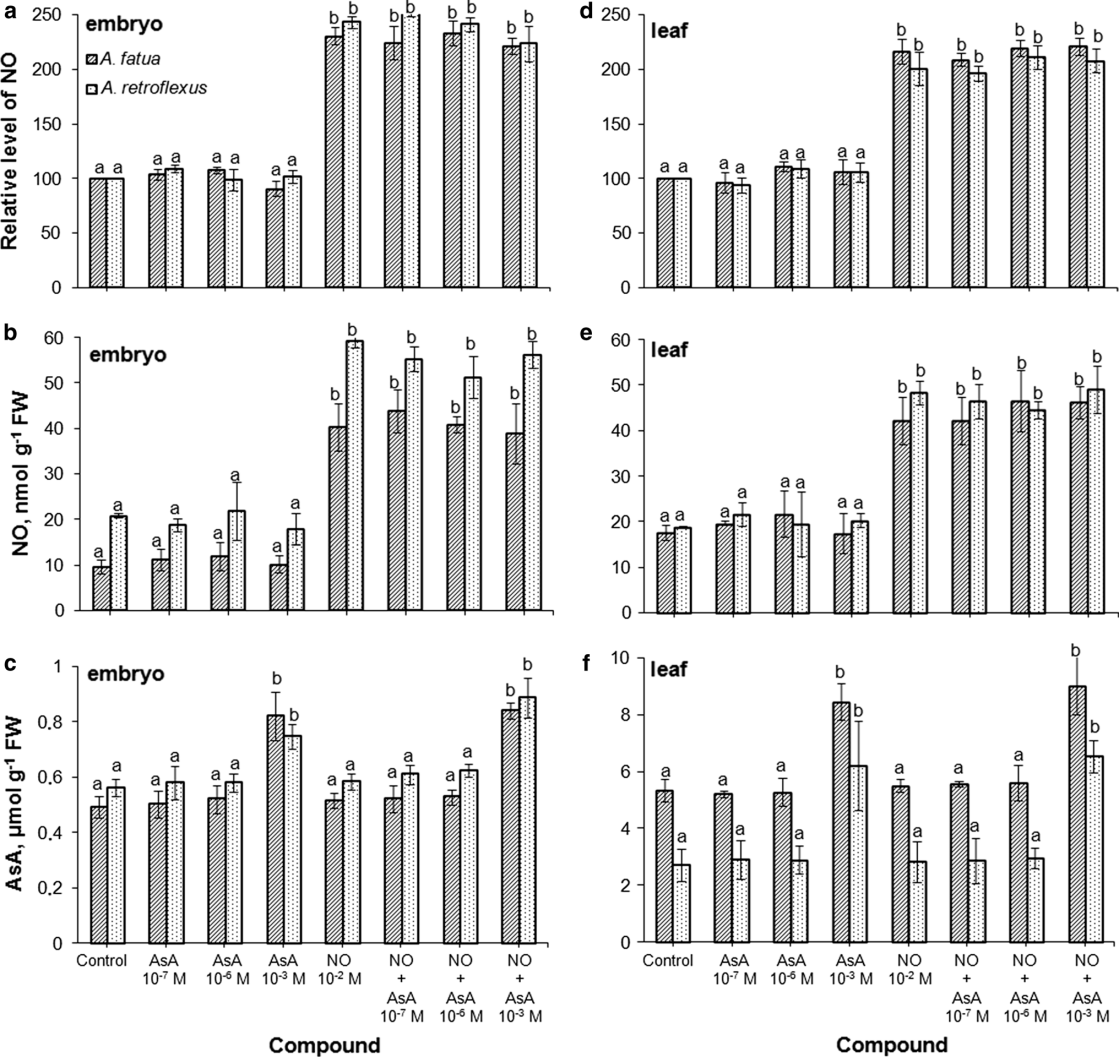
Effects of exogenous NO in the absence or presence of ascorbic acid (AsA) on the NO level as measured by FCM (**a**, **d**) or Griess reaction (**b**, **e**) and AsA content (**c**, **f**) in *A. fatua* and *A. retroflexus* embryos or leaves. Intracellular level of NO was measured by FCM after treatment with DAF-FM DA at 10 μM for 30 min. Fluorescence data (**a**, **d**) are expressed as mean fluorescence intensity (percentage of control). No autofluorescence was detectable when embryos or leaves were incubated without DAF-FM DA. Vertical bars indicate ± SD. One-way ANOVA with Duncan’s post hoc test was used to identify significant differences. Means with different letters (a–b) are significantly different (*P* < 0.05)

**Fig. 5:**
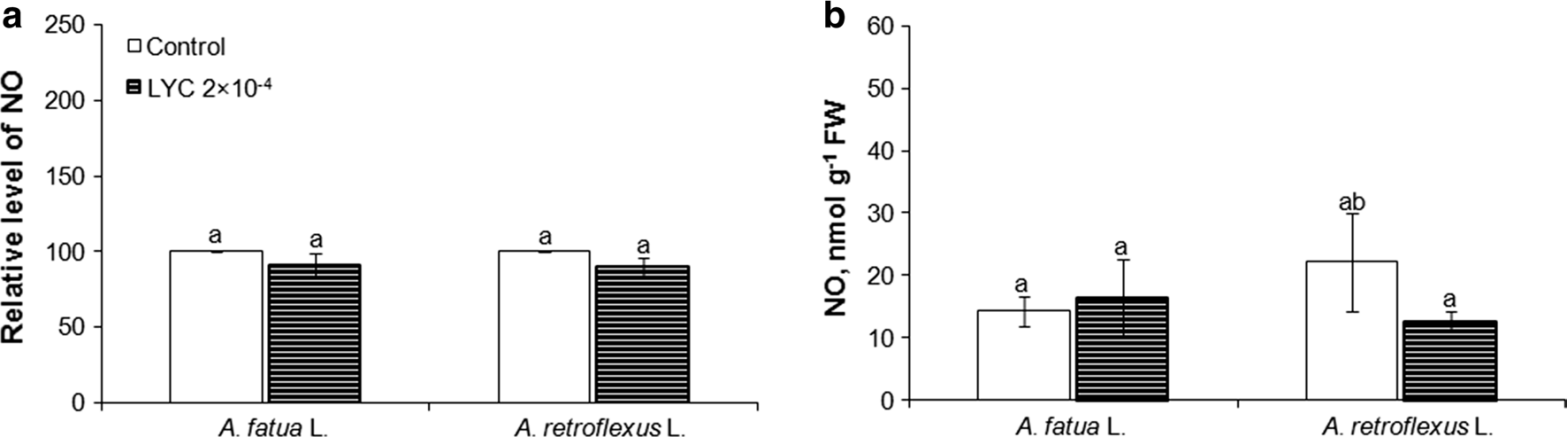
Effects of AsA biosynthesis inhibitor, lycorine (LYC) on the NO level as measured by FCM (**a**) or Griess reaction (**b**) in *A. fatua* and *A. retroflexus* embryos. Intracellular level of NO was measured by FCM after treatment with DAF-FM DA at 10 μM for 30 min. No autofluorescence was detectable when embryos or leaves were incubated without DAF-FM DA. Fluorescence data (**a**) are expressed as mean fluorescence intensity (percentage of control). Vertical bars indicate ± SD. One-way ANOVA with Duncan’s post hoc test was used to identify significant differences. Means with different letters (a–b) are significantly different (*P* < 0.05)

The fluorescence signal was also detected by FCM in cells of leaves of *A. fatua* and *A. retroflexus* only when they were treated with DAF-FM DA (Fig. 2c, d), that is, as in the case of embryos, no autofluorescence was found (not shown). Exogenous NO increased the mean fluorescence by a factor of 2 and 3.7 in leaves of *A. fatua* and *A. retroflexus*, respectively (Fig. 3c). On the other hand, cPTIO significantly decreased the fluorescence from both *A. fatua* and *A. retroflexus* leaves; the fluorescence intensity was decreased three times relative to untreated leaves. Ascorbic acid (AsA), applied to *A. fatua* and *A. retroflexus* embryos and leaves, did not affect the fluorescence signal related to NO (Fig. 4a, d). The fluorescence intensity in NO-treated embryos and leaves did not change after additional application of AsA. The AsA biosynthesis inhibitor, lycorine (LYC), applied to *A. fatua* and *A. retroflexus* embryos did not affect the fluorescence signal, either (Fig. 5a). The effect of mechanical wounding of *A. fatua*, *A. retroflexus,* and *M. truncatula* leaves on the fluorescence signal level was also determined. Immediately after wounding of *A. fatua* leaves, the intensity of the signal increased; the signal remained similar for the next 15 min, and until the end of the experiment was identical to that in the intact leaves (Fig. 7a). Wounding of *A. retroflexus* leaves also increased the intensity of the signal; the intensity decreased 15 min after the wounding, and was the same as in the intact leaves for up to 60 min. In the case of *M. truncatula,* wounding increased the fluorescence signal only after 30 min, and until the end of the experiment, the signal intensity was similar to that in the untreated leaves.

### Intracellular NO imaging

A real-time living cell imaging system was used to determine the intracellular location of NO in cell suspensions after application of DAF-FM DA. As expected, the fluorescence signal was non-detectable in *M. truncatula* cells in suspension not stained with DAF-FM DA, but staining with the dye did produce the signal (Fig. 1). In general, the fluorescence signal distribution in cells was heterogenous. The signal was mainly observed in the cell wall. In addition, an intense punctuated fluorescence signal was detected in the cytoplasm.

### NO level measured by the Griess assay

The presence of NO measured as a stable product of NO-to-nitrite reaction was found in embryos and leaves of *A. fatua* and *A. retroflexus* (Fig. 3b, d). Exogenous NO caused an about 2.8**-**fold increase in the NO content in embryos of both plants, compared to the NO levels in untreated embryos (Fig. 3b). The cPTIO treatment tended to reduce the NO content. NO was also measurable in the experiment involving leaves of *A. fatua* and *A. retroflexus*. Exogenous NO increased NO content in leaves of *A. fatua* and *A. retroflexus* by a factor of 2.5 and 2, respectively (Fig. 3d). cPTIO significantly decreased the NO content in leaves: the NO level in leaves of *A. fatua* and *A. retroflexus* was 1.8 and 2.7 times lower, respectively, compared to that in untreated leaves. AsA did not affect content of NO in embryos and leaves of *A. fatua* and *A. retroflexus* (Fig. 4b, e). In the next experiment, again, the NO levels in cells of embryos and leaves of the two species increased due to the exogenous NO treatment. The NO content in NO-treated embryos and leaves remained unchanged after an additional application of AsA. Lycorine (LYC) had no effect on the NO content in embryos of both species (Fig. 5b). Wounding of *A. fatua* leaves increased the NO content; 15 min after wounding the content was similar and later decreased to a level similar to that in intact leaves (Fig. 7b). Immediately after wounding of *A. retroflexus* leaves, the NO content increased and remained similar to that in intact leaves until the end of the experiment. The wounded leaves of *M. truncatula* showed the NO content higher than that in the intact leaves only 30 min after wounding.

### Ascorbic acid level

**Fig. 6:**
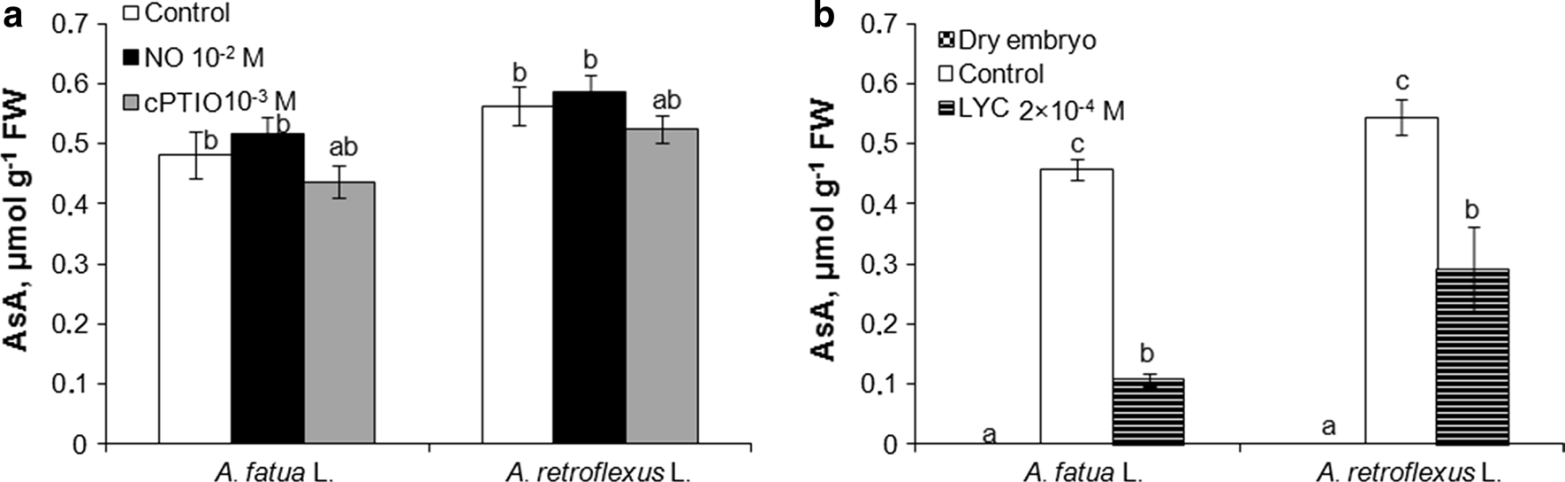
Effect of exogenous NO, cPTIO (**a**) or lycorine (LYC; **b**) on the ascorbic acid (AsA) content in *A. fatua* and *A. retroflexus* embryos. Embryos isolated from water-imbibed caryopses (**a**) were pretreated with NO or cPTIO for 1 h, or from LYC-imbibed caryopses (**b**) for 24 h were used for AsA determination. Vertical bars indicate ± SD. One-way ANOVA with Duncan’s post hoc test was used to identify significant differences. Means with different letters (a–c) are significantly different (*P* < 0.05)

**Fig. 7:**
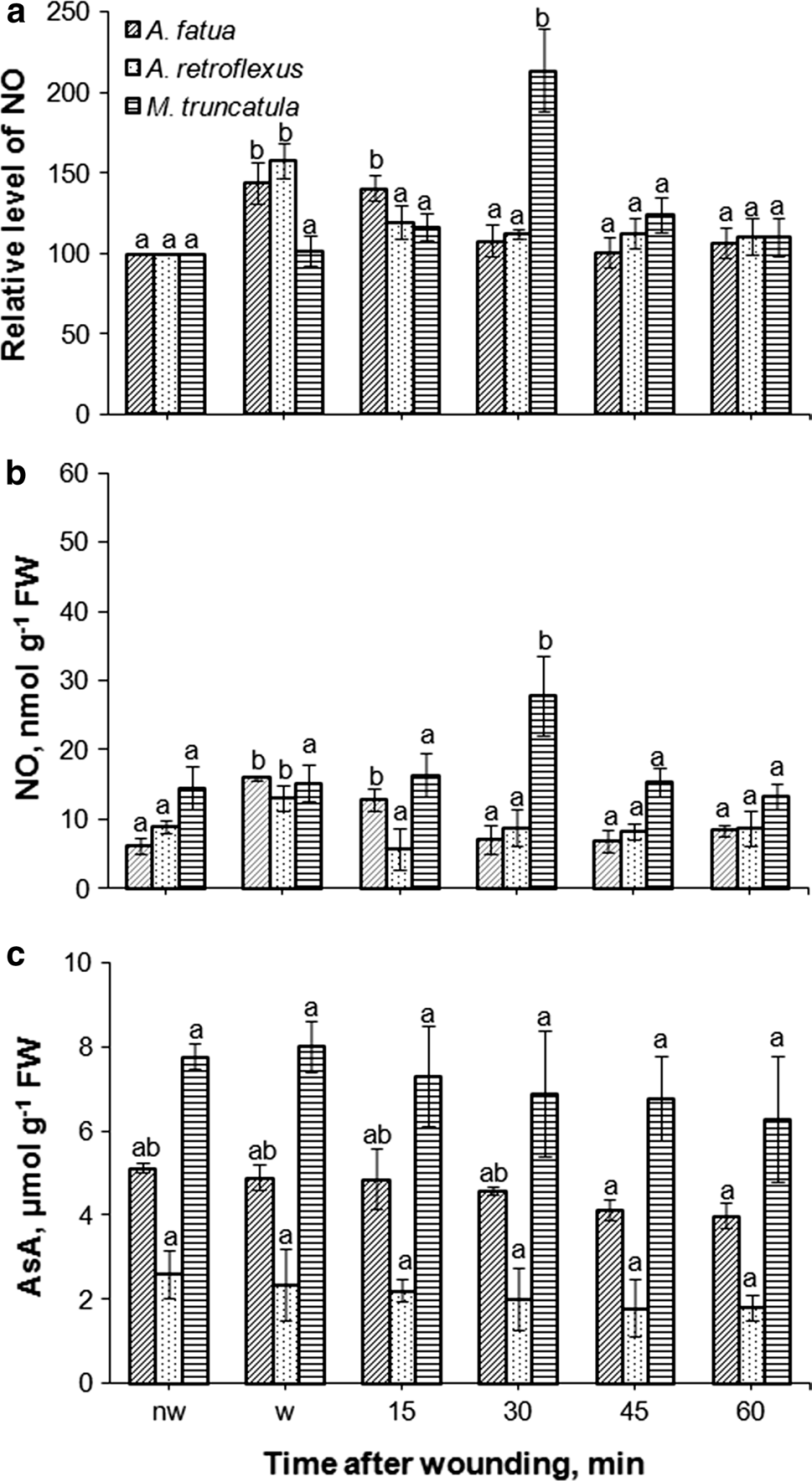
Effect of wounding on the NO level as measured by FCM (**a**) or Griess reaction (**b**) and AsA content (**c**) in *A. fatua*, *A. retroflexus* and *M. truncatula* leaves. Intracellular NO level was measured by FCM after treatment with DAF-FM DA at 10 μM for 30 min. No autofluorescence was detectable when embryos or leaves were incubated without DAF-FM DA. Nw, intact (not wounded); w, wounded. Fluorescence data (**a**) are expressed as mean fluorescence intensity (percentage of control). Vertical bars indicate ± SD. One-way ANOVA with Duncan’s post hoc test was used to identify significant differences. Means with different letters (a–b) are significantly different (*P* < 0.05)

AsA was not detected in embryos isolated from dry *A. fatua* caryopses and *A. retroflexus* seeds (Fig. 6b). AsA was detectable after 1 h of imbibition, the level remaining similar after incubation prolonged up to 24 h (Fig. 6a, b). Neither NO nor cPTIO affected the level of endogenous ascorbic acid in contrast to the effects of lycorine (LYC), which markedly suppressed the AsA level in embryos of *A. fatua* and *A. retroflexus.* The AsA treatment increased the compound’s level in embryos and leaves of both species only when applied at the highest concentration (Fig. 4c, f). The AsA increase due to the AsA treatment was at its highest in *A. retroflexus* leaves. Wounding did not affect the AsA level in leaves of *A. fatua, A. retroflexus* and *M. truncatula* (Fig. 7c).

## Discussion

### Optimization of flow cytometry methods for the detection and determination of NO production

Nitric oxide (NO) plays an important role as a signal in various plant processes and occurs at low concentrations (the rate of production 0.1–600 nmol g^−1^ FW h^−1^) (Neill et al. 2008), it is essential to use a highly sensitive method to measure it. Several methods with which to measure free NO or NO oxidation products in plants have been developed (Vitecek et al. 2008; Mur et al. 2011). In plant physiology, the fluorescent probes DAF-2 DA or DAF-FM DA have been most commonly used for NO detection by fluorescence microscopy and NO measurement by spectrofluorometry. Previously, DAF-2 DA (Strijdom et al. 2004; Tiscornia et al. 2009) and DAF-FM DA (Paul et al. 2011) were used in NO detection and determination by flow cytometry (FCM) only in human and animal cells such as human immunocells, cardiomyocytes and endothelial cells. To our knowledge, the present study is the first application of FCM to the determination of NO production in plant cells. The results obtained demonstrate the possibility of using FCM for the determination of the NO intracellular level in *M. truncatula* cells in suspension and in leaves, and also in cells of embryos and leaves of *A. fatua* and *A. retroflexus*. Detection of NO in cell suspension required incubation at 5 µM DAF-FM DA for 30 min (Fig. 1). Higher concentrations and a longer incubation time, up to 60 min, did not affect the fluorescence intensity, indicating that all NO molecules reacted with the dye used. The presence of NO in cell suspensions found with FCM was confirmed by the real-time living cell imaging system. The fluorescent dye concentration and/or treatment duration required that the NO level measurements be adjusted to the type of human/animal cells and kind of dye. Determination of NO in bovine aortic endothelial cells required incubation in DAF-2 DA at 10 µM for 1 h (Navarro-Antolin and Lamas 2001), 10 µM and 2 h being required for human peripheral blood monocultural cells (Tiscornia et al. 2009). To measure NO in suspensions of rat cardiomyocytes, 10 µM DAF-2 DA was used for up to 180 min (Strijdom et al. 2004). For the NO measurement in human endothelial cells, these were stained only with 1 µM of DAF-FM DA for 30 min (Paul et al. 2011). Our experiments showed staining with 10 µM DAF-FM DA for 30 min to be recommended for *A. fatua* and *A. retroflexus* embryos and leaves (Fig. 2). Since the signal intensity did not change for up to 60 min of incubation, the measurement can take between 30 and 60 min. The published results as well as the data obtained in this study show the need to adjust staining conditions to the biological material at hand. It should be emphasized that the methodology applied here does not differ much from that used previously in experiments with human or animal cells.

### NO production, measured by FCM, was confirmed by the Griess assay. The fluorescence signal is related to NO but not to AsA

It has been previously found that exogenous NO stimulates germination of dormant *Arabidopsis* (Bethke et al. 2004; Libourel et al. 2006) and *A. retroflexus* seeds (Kępczyński and Sznigir 2014; Kępczyński et al. 2017), indicating penetration of cells by NO. To confirm that FCM measures the NO level, embryos and leaves were treated with exogenous NO released from an acidified KNO_2_ (Yamasaki 2000), at a concentration which was previously used to treat *A. retroflexus* seeds to increase the level of the compound in cells (Kępczyński et al. 2017). The NO treatment markedly increased the fluorescence intensity in cells of both embryos and leaves (Figs. 2, 3a, c). Similarly, the NO treatment applied to apple embryos increased the DAF fluorescence measured by a fluorescence spectrophotometer (Gniazdowska et al. 2010). It can thus be concluded that, in all the cases mentioned above, the increase in the fluorescence intensity was associated with a higher NO level in cells resulting from the exogenous NO treatment. The NO scavenger, cPTIO, commonly used to study the role of endogenous NO in plants (Vitecek et al. 2008; Mur et al. 2011) was found to reduce the stimulatory effect of exogenous NO, ethylene and GA_3_ on germination of *A. retroflexus* seeds (Kępczyński et al. 2017). The application of cPTIO markedly reduced the fluorescence intensity in embryos and leaves of *A. fatua* and *A. retroflexus*, as measured by FCM (Figs. 2a, c, 3a, c), in *Arabidopsis* seeds, as measured by confocal laser scanning microscopy (Liu et al. 2009), and in apple embryos, as determined using a fluorescence spectrophotometer (Gniazdowska et al. 2010).

To validate the FCM as a method with which to determine the NO level in embryos and leaves, the Griess method was used to measure the NO concentration. Both methods produced comparable results; in both cases, exogenous NO increased the NO content in embryos and leaves, cPTIO decreasing it (Figs. 2, 3). The FCM’s advantage over the Griess assay is that it allows the intracellular NO level to be measured, while the Griess assay simultaneously measures the extra- and intracellular contents.

Since the previous experiments with animal cells showed that DAF-2 can react not only with NO to yield fluorescent DAF-2 triazole, but also with ascorbic acid (AsA) to generate compounds causing a similar fluorescence emission (Zhang et al. 2002), the fluorescence levels observed in *A. fatua and A. retroflexus* embryos and leaves after treatment with AsA alone or in combination with NO were also determined. The increasing endogenous AsA concentration by exogenous application of the compound (Fig. 4c, f) did not increase the fluorescence signal in comparison with the control, and did not increase the intensity of the signal caused by the NO treatment, either (Fig. 4a, d). It is thus evident that FCM detects NO, but not AsA. The lack of AsA effect on the NO production was also shown when the Griess reaction was used to determine NO (Fig. 4b, e). Furthermore, lycorine (LYC)—at the concentrations used—was found to inhibit the stimulatory effect of karrikinolide (KAR_1_) and GA_3_ on germination of dormant *A. fatua* caryopses (Cembrowska-Lech and Kępczyński 2016). As expected, it decreased the AsA levels in embryos of both *A. fatua and A. retroflexus* (Fig. 6b). However, it did not affect the signal intensity as measured by flow cytometry; the NO level as determined by the Griess reaction was not affected, either (Fig. 5). Moreover, both NO and cPTIO did not affect the AsA levels in embryos of both species (Fig. 6a), although the fluorescence signal and NO content as measured with the Griess method increased or decreased (Fig. 3a, b). Thus, the results described above, too, lend support to the conclusion that the fluorescence signal intensity, measured by FCM in our biological system, is related only to NO.

### Wounding affects the NO level, as measured by FCM and the Griess assay, but not the AsA content

Previously, a strong NO burst after wounding of *Arabidopsis* leaves was found (Huang et al. 2004). To find out whether FCM can be used to measure the NO production due to injury, the fluorescence signal was measured in intact and mechanically wounded leaves of three plant species. Wounding increased the fluorescence signal in comparison with intact leaves of all the species used, the AsA levels not being affected (Fig. 7a, c). Likewise, results produced by the Griess method showed increased NO levels due to wounding (Fig. 7b). They were thus again in good agreement with the FCM data (Fig. 7a), indicating the usefulness of FCM for the NO measurement.

To sum up, the data obtained in this study, which involved manipulating the endogenous AsA concentration by applying AsA or its biosynthesis inhibitor, showed—in contrast to experiments with animal cells (Zhang et al. 2002)—that the compound did not affect the fluorescence signal. Thus, FCM can be applied to measure NO in cells of embryos and leaves of the species used in this study despite the presence of AsA. It should be emphasized that the AsA level in the experiments described in this paper was lower than 10 µmol g^−1^ FW, while more than 5 mM AsA is required to elicit a detectable fluorescence in plants (Planchet and Kaiser 2006).

In DAF-2-DA-treated tobacco cells, hydrogen peroxide was found to produce a similar signal, measured by fluorimetry, to that of NO. Therefore, caution was recommended in the use of DAF-2-based simple fluorescence measurements or fluorescence imaging for safe determination of the NO production; it was suggested that at least two methods, based on different reactions, be used to determine NO in vivo (Rümer et al. 2012). Despite the objections regarding the use of diaminofluoresceins for the measurement of NO, these dyes are still used to detect or determine production of this gas in plants using a fluorescence microscopy or fluorimetry (Krasuska et al. 2017; Zhao et al. 2017). However, the data presented in the present paper indicate that FCM with DAF-FM DA can be applied to determine the intracellular NO production in cells of embryos and leaves of the species used, since the applicability of the method to quantification of the NO production was confirmed by the Griess assay.

In summary, application of FCM with DAF-FM DA provides a new opportunity to use a simple and rapid method for the detection and determination of intracellular NO production *M. truncatula* cells in suspension and leaves as well as in embryos and leaves of *A. fatua* and *A. retroflexus* and probably other plant tissues. It is also worth underlining that the advantage of the method lies in its allowing to measure the intracellular content of the gas in contrast to several other methods. However, to be on the safe side, it is recommended to use, in parallel, both FCM with DAF-FM DA and the Griess assay or other methods. In some earlier studies, results obtained with spectrofluorometry were confirmed with HPLC, electron paramagnetic resonance or chemiluminescence (Mur et al. 2011).

#### Author contribution statement

JK initiated the research, interpreted results and wrote the final version of the manuscript. DC conducted experiments and statistical analysis, and wrote the first preliminary draft of the manuscript. All authors read and approved the manuscript.

## Abbreviations

cPTIO: Carboxy-PTIO, 2-(4-carboxyphenyl)-4,4,5,5-tetramethylimidazoline-1-oxyl-3-oxide
DAF-FM DA: 4-amino-5-methylamino-2′,7′-difluorofluorescein diacetate
FCM: Flow cytometry
NO: Nitric oxide
